# Moxibustion and other acupuncture point stimulation methods to treat breech presentation: a systematic review of clinical trials

**DOI:** 10.1186/1749-8546-4-4

**Published:** 2009-02-27

**Authors:** Xun Li, Jun Hu, Xiaoyi Wang, Huirui Zhang, Jianping Liu

**Affiliations:** 1 Centre for Evidence-Based Chinese Medicine, Beijing University of Chinese Medicine, Beijing, PR China; 2 Centre for the History of Medicine, Peking University, Beijing, PR China; 3 School of Preclinical Medicine, Beijing University of Chinese Medicine, Beijing, PR China; 4 National Research Centre in Complementary and Alternative Medicine (NAFKAM), University of Tromsø, Tromsø, Norway

## Abstract

**Background:**

Moxibustion, acupuncture and other acupoint stimulations are commonly used for the correction of breech presentation. This systematic review aims to evaluate the efficacy and safety of moxibustion and other acupoint stimulations to treat breech presentation.

**Methods:**

We included randomized controlled trials (RCTs) and controlled clinical trials (CCTs) on moxibustion, acupuncture or any other acupoint stimulating methods for breech presentation in pregnant women. All searches in PubMed, the Cochrane Library (2008 Issue 2), China National Knowledge Information (CNKI), Chinese Scientific Journal Database (VIP) and WanFang Database ended in July 2008. Two authors extracted and analyzed the data independently.

**Results:**

Ten RCTs involving 2090 participants and seven CCTs involving 1409 participants were included in the present study. Meta-analysis showed significant differences between moxibustion and no treatment (RR 1.35, 95% CI 1.20 to 1.51; 3 RCTs). Comparison between moxibustion and knee-chest position did not show significant differences (RR 1.30, 95% CI 0.95 to 1.79; 3 RCTs). Moxibustion plus other therapeutic methods showed significant beneficial effects (RR 1.36, 95% CI 1.21 to 1.54; 2 RCTs). Laser stimulation was more effective than assuming the knee-chest position plus pelvis rotating. Moxibustion was more effective than no treatment (RR 1.29, 95% CI 1.17 to 1.42; 2 CCTs) but was not more effective than the knee-chest position treatment (RR 1.22, 95% CI 1.11 to 1.34; 2 CCTs). Laser stimulation at *Zhiyin *(BL67) was more effective than the knee-chest position treatment (RR 1.30, 95% CI 1.10 to 1.54; 2 CCTs,).

**Conclusion:**

Moxibustion, acupuncture and laser acupoint stimulation tend to be effective in the correction of breech presentation.

## Background

Breech presentation (opposite direction of the normal position of the foetus) is common in the mid-trimester of pregnancy, with the incidence of breech decreasing as the pregnancy approaches term. The incidence of breech presentation at term is reported to be 4% [1]. Women with breech presentation face serious problems if delivering vaginally. Breech presentation may arise from placenta praevia, multiple gestation, uterine abnormalities, poor uterine tone, pre-maturity or unknown causes, and is associated with primigravidae, older mothers, babies that are small for gestational age and female babies [2].

Caesarean section is often recommended for pregnant women with breech presentation who may otherwise prefer natural deliveries. Caesarean section does have distinct disadvantages including increased risks of maternal urinary tract infection, haemorrhage, wound infection and scar dehiscence or uterine rupture during subsequent labour [3].

Some conventional non-surgical therapies for breech presentation are available, such as the knee-chest position treatment and external manual cephalic version. However, knee-chest position is difficult to adopt and likely to cause inadequate compliance, whereas external cephalic version is much more complex, potentially dangerous, time consuming and expensive [4].

Moxibustion is a traditional method of burning moxa sticks (usually made from herbal preparations containing *Artemisia vulgaris*) near an acupoint to cause a warm and painless sensation [5]. In China, moxibustion on *Zhiyin *(BL67) point has long been used to correct abnormal foetal position and is widely used to correct breech presentation in obstetrics. Many clinical studies on this method were carried out and published in academic journals including JAMA [6]. Moxibustion and other acupoint stimulation methods such as acupuncture and laser stimulation were found to be effective to treat breech presentation.

The present systematic review aims to evaluate the efficacy and safety of moxibustion and other acupoint stimulation methods to treat breech presentation in pregnant women.

## Methods

### Databases and search strategy

Two authors (JH and XL) searched the China National Knowledge Infrastructure (CNKI) (1979–2008), Chinese Scientific Journal Database (VIP) (1989–2008), WanFang Database (WanFang) for Chinese Publications (1985–2008), PubMed (1966–2008), the Cochrane Library (Issue 2, 2008) and Traditional Chinese Medicine Database System. The last search was in July 2008. The search terms included 'abnormal foetal position (*taiwei buzheng*)', 'breech presentation *(tunwei)*', 'correction/conversion (*zhuantai*)', 'correct abnormal foetal presentation (*jiaozheng taiwei*/*jiuzheng taiwei*)', 'moxibustion', 'acupuncture', 'pregnancy', 'acupoint stimulation', '*Zhiyin*' and 'laser'. We manually retrieved some recognized articles not available electronically and also performed additional searches to identify potentially eligible trials from the retrieved studies and reviews in the electronic databases.

### Inclusion criteria

We included randomized controlled trials (RCTs) and non-randomized controlled clinical trials (CCTs) on moxibustion and/or other acupoint stimulation methods. There was no restriction on the race or gestation of participants, publication type or language. We excluded case-control studies, case series, case reports, non-clinical studies and trials to compare different acupoint stimulation methods.

### Study selection and data extraction

Three authors (XL, XYW and HRZ) selected studies according to the inclusion criteria. The paper titles and abstracts were read and assessed for their eligibility and relevance. The full texts of related papers were retrieved and reviewed based on the inclusion and exclusion criteria of the studies. We were not blinded to the names of the authors, institutions or journals of the published studies.

Two authors (XYW and HRZ) extracted the data independently using a structured data extraction form and another author (XL) verified the extracted data. Any discrepancies were discussed and consensus was reached. The extracted data included demographic data, quality of trial design, inclusion and exclusion criteria, interventions and results.

In the case of missing data in the included studies, we contacted the original investigators and conducted the intention to treat analysis (ITT).

### Quality assessment

Three authors (XYW, HRZ and JH) assessed the quality of each trial independently, according to the Cochrane handbook [7] and CONSORT statement for reporting RCTs [8,9], A generic grading system [10] was applied to the included RCTs and CCTs as follows:

#### A (good)

Studies in this category have the least biases and their results are considered valid. These studies have (1) clear description of the population, setting, interventions and comparison groups; (2) appropriate measurement of outcomes; (3) appropriate statistical and analytical methods; (4) no reporting errors; (5) less than 20 percent dropouts; (6) clear reporting of dropouts; and (7) appropriate consideration and adjustment for potential confounders.

#### B (fair)

Studies in this category are susceptible to some degrees of biases that are not sufficient to invalidate the results. These studies may have sub-optimal adjustments for potential confounders and may also lack certain information that is needed to assess limitations and potential problems.

#### C (poor)

Studies in this category have significant biases which may invalidate the results. These studies may have critical flaws in design, analysis and/or reporting, missing information and/or discrepancies in reporting. For instance, these studies either do not consider potential confounders or do not make adjustments for them appropriately.

The studies graded between B (fair) and C (poor) were reviewed and graded again by other authors. Consensus was reached through discussion. It should be noted that this summary quality grading system evaluates and grades the studies within their own design strata and does not attempt to assess the comparative validity of studies across different designs. Thus, the assessors and users should be cognizant of the study design when interpreting the methodological quality grade of a study.

### Data analysis

Review Manager Software 4.2.7 provided by the Cochrane Collaboration was used for data analysis. Dichotomous data were expressed as a risk ratio (RR) with a provision of 95% confidence interval (CI). Meta-analysis was performed if experimental intervention and control intervention were the same or similar, such as moxibustion versus knee-chest position. The statistical heterogeneity was analyzed and presented when I square (I^2^) is over 50% or *P *< 0.1 as significant. Random effect model was used for the meta-analysis if there was significant heterogeneity (I^2 ^> 50%) and fixed effect model was used when the heterogeneity was not significant (I^2 ^< 50%) [11].

## Results

### Description of included studies

**Figure 1:**
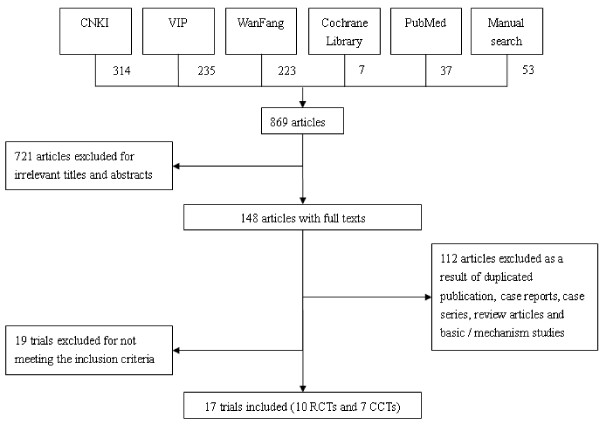
**Process of trial identification and selection**.

In this review, a total of 869 studies were screened out of which 148 studies with full texts were retrieved for selection according to the inclusion and exclusion criteria. A total of 131 papers were excluded, out of which 112 papers were duplicate publications, case reports, case series, review articles, basic research or mechanism studies, and 19 studies did not meet the inclusion criteria in terms of participants, interventions or outcomes (Additional file 1). As a result, 17 clinical trials including ten RCTs [6,12-20] and seven CCTs [21-27] were reviewed (Figure 1). In addition, two systematic reviews were identified. One of them is a Cochrane review which covered three trials published in 2005 [28] and another review covered six trials published in 2008 [29]. Three trials were conducted in Italy [14,15,24], one in Japan [22] and the remaining 13 in China. Six trials were published in English [6,14,15,17,22,24] and three trials had more than two arms.

Among the included trials, ten RCTs and seven CCTs involved 2090 and 1409 pregnant women respectively. These trials investigated moxibustion (13 trials), acupuncture (2 trials), electro-acupuncture (1 trial), laser stimulation (2 trials) or ear acupuncture (1 trial) on acupoints and comparisons with no treatment (7 trials), knee-chest position (10 trials), raising buttocks method (2 trials) or throwing breech (1 trial) for the correction of breech presentation (Table 1). Among all the included trials, 14 trials [6,12-19,21,22,24-26] used ultrasound to confirm the diagnosis of breech presentation. Four trials reported adverse effects.

**Table 1:** Characteristics of included trials of moxibustion, acupuncture and other acupoint stimulation for breech presentation

**Study ID**	**Design**	**Sample size**	**Age (year)** **(Rx/control)**	**Gestation age (week)** **(Rx/control)**	**Intervention**	**Control**	**Outcomes**
Cardini 2005 [14]	RCT	123	31	33	moxibustion on *Zhiyin*	no treatment	cephalic presentation
Cardini 1998 [6]	RCT	260	25.5/25.2	33/33	moxibustion on *Zhiyin*	no treatment	number of cephalic presentation, foetal activity; number and causes of caesarean deliveries, spontaneous and induced vaginal deliveries, Apgar score, adverse events
Huang 1990 [20]	RCT	587	NA	28–32	moxibustion on *Zhiyin*	no treatment or knee-chest position	cephalic presentation
Yang 2006 [13]	RCT	206	23.1–30.3	28–34	moxibustion on *Zhiyin *plus knee-chest position	knee-chest position	cephalic presentation, adverse events
Lin 2002 [18]	RCT	122	21–38	30–37	moxibustion on *Zhiyin*	knee-chest position	cephalic presentation
Peng 2006 [12]	RCT	80	21–36	30–34	moxibustion on *Zhiyin*	knee-chest position	cephalic presentation
Chen 2004 [16]	RCT	142	22–38/22–39	30–34/30–34	moxibustion on *Zhiyin *plus raising buttocks method	raising buttocks method	cephalic presentation
Habek 2003 [17]	RCT	67	22 ± 3.1/23 ± 1.3	34–37	acupuncture on *Zhiyin*	no treatment	cephalic presentation
Neri 2004 [15]	RCT	240	31.7+4.7/30.1+3.6	33.5+0.6/33.7+0.7	acupuncture plus moxibustion on *Zhiyin*	no treatment	cephalic presentation, adverse events
Ye 1998 [19]	RCT	263	28.35	28–36/28–33	laser stimulation on *Zhiyin*	knee-chest position plus pelvis rotating	cephalic presentation
Liang 2004 [21]	CCT	320	NA	28	moxibustion on *Zhiyin*	knee-chest position	cephalic presentation
Xiong 1991 [26]	CCT	60	20–28/20–28	32–36/32–36	moxibustion on *Zhiyin*	knee-chest position	cephalic presentation
Wu 1995 [23]	CCT	820	20–37	20–37	moxibustion on *Zhiyin *plus raising buttocks method or laser stimulation on *Zhiyin*	raising buttocks method or knee-chest position	cephalic presentation
Jiang 1993 [25]	CCT	382	20–38	30–40	laser stimulation on *Zhiyin*	knee-chest position	cephalic presentation
Qin 1989 [27]	CCT	150	NA	30–37	ear acupuncture	knee-chest position	cephalic presentation
Kanakura 2001 [22]	CCT	548	28.4	28 (minimal)	moxibustion or electro-acupuncture	no treatment	cephalic presentation
Cardini 1993 [24]	CCT	41	20–37	22–31	moxibustion on *Zhiyin*	no treatment	cephalic presentation

### Methodological qualities

Three RCTs [6,14,15] published in English were rated as A (good). One RCT published in Chinese [12], which met the inclusion criteria, was rated as C (poor) and the remaining RCTs were rated as B (fair). One CCT [25], which met the inclusion criteria, was rated as C (poor) and the remaining CCTs were all rated as B (fair).

### Efficacy of the interventions

#### Moxibustion and/or acupuncture on Zhiyin versus no treatment

Three RCTs [6,14,20] found significant differences between moxibustion group and no treatment group (RR 1.35, 95% CI 1.20 to 1.51). Another RCT [17] did not find significant benefit in acupuncture group. Significant benefit was found in acupuncture plus moxibustion group in an RCT [15].

ITT analysis was performed on three trials, however, the effect size and direction of correction rate remained the same [6,14,15].

Two CCTs [22,24] found significant benefit in moxibustion group and a CCT [22] showed significant benefit in acupuncture group (Table 2).

**Table 2:** Efficacy of moxibustion, acupuncture or other acupoint stimulations for the correction of breech presentation

	Treatment (n/N, %)	Control (n/N, %)	Relative benefit (95% CI)	*P *value
**Randomized controlled trial**				
*Moxibustion on Zhiyin vs no treatment*				
Cardini 1998 [6]	98/129 (76.0)	62/106 (58.5)	1.30 [1.08, 1.57]	0.006
Huang 1990 [20]	150/193 (77.7)	106/200 (53.0)	1.47 [1.26, 1.71]	< 0.00001
Cardini 2005 [14]	22/65 (33.8)	21/58 (36.2)	0.93 [0.58, 1.51]	0.78
*Meta-analysis*	270/387 (69.8)	189/364 (51.9)	1.35 [1.20, 1.51]	< 0.00001
				
*Acupuncture on Zhiyin vs no treatment*				
Habek 2003 [17]	31/34 (91.2)	26/33 (78.8)	1.16 [0.94, 1.42]	0.16
*Acupuncture plus moxibustion on Zhiyin vs no treatment*				
Neri 2004 [15]	61/120 (50.8)	43/120 (35.8)	1.42 [1.05, 1.91]	0.02
*Moxibustion on Zhiyin vs knee-chest position*				
Huang 1990 [20]	150/193 (77.7)	115/194 (59.3)	1.31 [1.14, 1.51]	0.0001
Lin 2002 [18]	58/63 (92.1)	31/59 (52.5)	1.75 [1.36, 2.26]	< 0.0001
Peng 2006 [12]	16/40 (40.0)	20/40 (50.0)	0.80 [0.49, 1.31]	0.73
Meta-analysis*	224/296 (75.7)	166/293 (56.7)	1.30 [0.95, 1.79]	0.1
				
*Moxibustion on Zhiyin plus raising buttocks method vs raising buttocks method*				
Chen 2004 [16]	67/73 (91.8)	36/69 (52.2)	1.76 [1.39, 2.23]	0.02
*Moxibustion on Zhiyin plus knee-chest position vs knee-chest position*				
Yang 2006 [13]	90/103 (87.4)	77/103 (74.8)	1.17 [1.02, 1.34]	< 0.00001
*Meta-analysis*	157/176 (89.2)	113/172 (65.7)	1.36 [1.21, 1.54]	< 0.00001
				
*Laser stimulation on Zhiyin vs knee-chest position plus pelvis rotating*				
Ye 1998 [19]	108/133 (81.2)	73/130 (56.2)	1.45 [1.22, 1.72]	< 0.0001
				
**Controlled clinical trial**				
*Moxibustion on Zhiyin vs no treatment*				
Kanakura 2001 [22]	123/133 (92.5)	165/224 (73.7)	1.26 [1.15, 1.38]	< 0.00001
Cardini 1993 [24]	16/23 (69.6)	7/18 (38.9)	1.79 [0.94, 3.39]	0.07
*Meta-analysis*	139/156 (89.1)	172/242 (71.1)	1.29 [1.17, 1.42]	< 0.00001
				
*Acupuncture on Zhiyin vs no treatment*				
Kanakura 2001 [22]	171/191 (89.5)	160/217 (73.7)	1.21 [1.11, 1.33]	< 0.001
*Moxibustion on Zhiyin vs knee-chest position*				
Liang 2004 [21]	144/160 (90.0)	126/160 (78.8)	1.14 [1.04, 1.26]	0.007
Xiong 1991 [26]	29/30 (96.7)	16/30 (53.3)	1.81 [1.29, 2.55]	0.003
Meta-analysis*	173/190 (91.1)	142/190 (74.7)	6.31 [0.63, 63.17]	0.12
				
*Laser stimulation on Zhiyin vs knee-chest position*				
Jiang YH 1993 [25]	218/278 (78.4)	66/104 (63.5)	1.24 [1.05, 1.45]	0.003
Wu 1995 [23]	314/432 (72.7)	25/51 (49.0)	1.48 [1.11, 1.97]	0.0007
*Meta-analysis*	532/710 (74.9)	91/155 (58.7)	1.30 [1.10, 1.54]	< 0.00001
				
*Ear acupuncture vs knee-chest position*				
Qin 1989 [27]	84/99 (84.8)	26/39 (66.7)	1.27 [1.00, 1.61]	0.02
*Moxibustion on Zhiyin plus raising buttocks method vs raising buttocks method*				
Wu 1995 [23]	103/192 (53.6)	76/145 (52.4)	1.02 [0.84, 1.25]	0.82
*Laser stimulation on Zhiyin vs raising buttocks method*				
Wu 1995 [23]	314/432 (72.7)	76/145 (52.4)	1.39 [1.18, 1.64]	0.00001

*Random effect model

#### Moxibustion, laser stimulation or ear acupuncture versus knee-chest position

Three RCTs [12,18,20] published in Chinese found no significant difference between moxibustion and knee-chest position (RR 1.30, 95% CI 0.95 to 1.79), analyzed in a random effect model with significant heterogeneity (I^2 ^= 77.0%). However, a meta-analysis showed positive results (RR 1.33, 95% CI 1.18 to1.50), analyzed in a fixed effect model.

Two CCTs [21,26] published in Chinese showed significant benefit in moxibustion compared with knee-chest position, analyzed in a fixed effect model (RR 3.36, 95% CI 1.87 to 6.05), while they showed no significant benefit, analyzed in a random effect model (RR 6.31, 95% CI 0.63 to 63.17, significant heterogeneity I^2 ^= 77.7%). One CCT [27] on ear acupuncture and knee-chest position found significant benefit in ear acupuncture group.

Two CCTs [23,25] on laser stimulation found significant benefit compared with knee-chest position (Table 2).

#### Moxibustion plus other interventions versus other interventions

An RCT [16], which investigated moxibustion on *Zhiyin *plus raising buttocks method versus raising buttocks method alone, showed significant benefit in the combination treatment group. Another RCT [13], which investigated moxibustion on *Zhiyin *plus knee-chest position versus knee-chest position alone, showed significant benefit in the combination treatment group. A meta-analysis of the two RCTs showed significant benefit in favour of the combination treatment. A CCT [23] compared moxibustion plus raising buttocks method with raising buttocks method alone but did not find significant difference between the two groups (Table 2).

#### Laser stimulation on Zhiyin versus other interventions

An RCT [23] on laser stimulation plus knee-chest position demonstrated significant benefit compared to pelvis rotating treatment which is an exercise to rotate a pregnant woman's pelvis for the correction of breech presentation.

A CCT [25] compared laser stimulation with raising buttocks method and showed significant benefit in laser stimulation group (Table 2).

### Publication biases

Among the comparative trials, the maximal number of trials in one outcome was three. Due to the low number of trials, no meaningful funnel plots could be produced.

#### Safety

Four trials reported outcomes of adverse events unrelated to moxibustion treatment. Cardini and Weixin [6] reported two premature births and four preterm premature rupture of membranes (PPROM) in the treatment group among 129 participants, while three premature births, one intrauterine foetal death and 12 PPROM were reported in the control group. Cardini *et al. *[14] reported two cases of preterm deliveries, one of which was due to PPROM.

## Discussion

From the findings of the present study, moxibustion, acupuncture and other acupoint stimulation appear to be effective in the correction of breech presentation. However, the number of available trials was insufficient for us to draw a confident conclusion.

In both RCTs and CCTs, moxibustion showed significant favourable differences in comparison with no treatment. However, meta-analysis of both the RCTs and CCTs comparing moxibustion with knee-chest position showed non-significant differences in a random effect model due to a highly heterogeneity. The results were positive in a fixed effect model, which should be interpreted with caution.

To investigate the efficacy of knee-chest position in comparison with no treatment or placebo, we searched PubMed and identified a Cochrane systematic review of three RCTs [28]. This review did not find adequate evidence to support that moxibustion or knee-chest position had significant benefits in comparison with no treatment. However, our findings agree with a recently published systematic review of six RCTs and three cohort studies [29] suggesting that moxibustion and other acupuncture-type interventions at acupoint BL67 are effective in the correction of breech presentation and that the methodological quality of the available trials was limited. An ongoing multi-centre randomized trial may provide further evidence for the efficacy [30].

No biological synergistic actions have been suggested between moxibustion and other interventions such as knee-chest position or raising buttocks method; thus, these interventions may be independent from each other. When moxibustion plus another intervention shows significant beneficial effects compared with the respective non-moxibustion intervention, we may assume that the differences are caused by moxibustion. Under this assumption, we combined the trials with similar study designs [13,16] in our meta-analysis.

We included in the present review both randomized and non-randomized trials because many trials carried out in China are non-randomized which may provide supplementary evidence to randomized trials [31].

Double blinding was not practised in these trials as it is not practical to mask the practitioners and/or the patients during moxibustion and other acupoint stimulation interventions lack a suitable placebo. Outcomes for the correction of breech presentation were determined objectively by ultrasound.

The effectiveness of moxibustion may vary depending on participants' culture background, belief, preference and expectation as evidenced in two RCTs [6,14]. It should also be noted that breech presentation was corrected spontaneously at about 50% in the non treatment groups. Thus, these factors should be taken into consideration in designing clinical trials. Incorporating qualitative research into clinical trials may help interpret research findings [32].

Further randomized trials are warranted, in which several aspects should be addressed, such as study settings, patient preferences and expectations (qualitative research), characteristics of the pregnant women (e.g. age, ethnic group, term of pregnancy), a consensus protocol of the intervention, and clinical and end-point outcomes. Trials should be reported according to the CONSORT Statement [33].

## Conclusion

From the findings of the present study, moxibustion, acupuncture and laser stimulation at acupoints showed beneficial effects for the correction of breech presentation. However, studies such as multi-centre trials are warranted.

## Abbreviations

CCTs: controlled clinical trials; CI: confidence interval; CNKI: China National Knowledge Infrastructure; I^2^: I square; ITT: intention to treat analysis; PPROM: preterm premature rupture of membranes; RCTs: randomized controlled trials; RR: risk ratio; VIP: Chinese Scientific Journal Database; WanFang: WanFang Database.

## Competing interests

The authors declare that they have no competing interests.

## Authors' contributions

XL and JPL conceived the review topic and drafted the manuscript. JPL revised the manuscript and provided perspectives on methodological issues. JH and XL performed the electronic and manual searches respectively, and conducted study selection, data extraction and analysis, and quality assessment. XYW and HRZ performed the manual searches, data extraction and quality assessment. All authors read and approved the final version of the manuscript.

## Supplementary Material

Additional file 1 **Clinical trials excluded from the present review**. The table provides the bibliographic information of the clinical trials excluded from the present review and the reasons for exclusion. Click here for file
