# Supplementary material for: Moxibustion and other acupuncture point stimulation methods to treat breech presentation: a systematic review of clinical trials
Source: Chin Med. 2009 Feb 27;4:4. doi: 10.1186/1749-8546-4-4 (PMC2663768; doi:10.1186/1749-8546-4-4)
Supplement: Additional file 1 — Clinical trials excluded from the present review. The table provides the bibliographic information of the clinical trials excluded from the present review and the reasons for exclusion. [file 1749-8546-4-4-S1.doc]

Clinical trials excluded from the present review

| **Reference** | **Reasons for exclusion** |
| --- | --- |
| Chai YH: **40 cases of rotator by finger press combined with moxibustion on Zhiyin(BL67).** *Shanghai Zhenjiu Zazhi* 1996, **15:**22. | RCT comparing finger pressure (*Zhiqie*) combined with moxibustion and acupuncture combined with moxibustion to correct breech presentation |
| Chen Y: **Moxibustion on Zhiyin for abnormal fetal position in 80 cases.** *Shaanxi Zhongyi Zazhi* 2007, **28:**334-335. | RCT involving participants of both breech and shoulder presentations |
| Du YH: **Intergrative Chinese-Western medicine for abnormal fetal position in 50 cases.** *Xiandai Zhongxiyi Jiehe Zazhi* 2005, **14:**2727. | RCT involving participants of both breech and shoulder presentations |
| Fang ML: **Analyzing 154 cases corrected foetal circulation with pelvic presentation**. *Hebei Yixue* 2006, **12:**257-259. | CCT comparing moxibustion combined with knee-chest position and external foetal position transition |
| Lai ZJ: **Effectiveness observation on ginger moxibustion for correction of fetal position in 80 cases.** *Xin Zhongyi* 2003, **35:**59. | RCT comparing time points on same intervention type |
| Li QH, Wang L: **Clinical observation on correcting malposi- tion of fetus by electro-acupuncture.** *J Tradit Chin Med* 1996, **16**(4):260-262. | RCT involving pregnant women with breech, transverse and occipitoposterior positions, outcome not reported separately |
| Li Y: **Hanging moxibustion on Sanyinjiao for abnormal fetal position in 60 cases.** *Zhongyi Waizhi Zazhi* 2006, **15:**47-48. | RCT comparing moxibustion at acupoints *Sanyinjiao* and *Zhiyin* |
| Liang JL, Chen SR, Li YP: **Comparative Analysis of Moxibustion at Zlliyin Acupoint and Knee-chest Posture in Correcting Breech Presentation，Report of 320 Cases.** *Huaxia Yixue* 2004, **17:**11-12. | CCT involving participants of both breech and shoulder presentations |
| Liu HC, Sun GX, Ma XC: **Clinical observation of moxibustion on Zhiyin(BL 67) combined with side-lying position for correcting breech presentation**. *Zhongguo Shequ Yishi Zazhi* 2001, **12:**40-41. | RCT involving moxibustion in both groups |
| Liu JL, Jin LY: **Effectiveness observation on topical application by ginger on Zhiyin for correction of abnormal fetal position.** *Zhongguo Xiandai Yisheng* 2007, **45:**157. | CCT involving ginger topical application controlled with knee-chest position plus moxibustion on *Zhiyin* |
| Niu XX, Niu Q: **Curative effect observation of moxibustion on Zhiyin acupoint for treating difficult labor.** *Shanghai Zhenjiu Zazhi* 2006; **6:**29. | RCT involving moxibustion to treat difficult labour |
| Wang AB, Xia XQ: **Cowherb pressing for abnormal breech presentation in 68 cases.** *Zhongguo Minjian Liaofa* 2002, **10:**29-30. | RCT involving participants of both breech and shoulder presentations |
| Wu YC: **Warm acupuncture for fetal position correction and experiment observation.** *Shanghai Zhenjiu Zazhi* 1994, **13:**150. | RCT comparing warm acupuncture on acupoints *Sanyinjiao* and *Zhiyin* |
| Xu FL, Zhu GM, Li YM: **Clinical observation of correcting breech presentation by moxibustion and self-correcting method for breech presentation.** [*Hei*](http://ckrd.cnki.net/GRID20/Navi/Bridge.aspx?LinkType=BaseLink&DBCode=cjfd&TableName=cjfdbaseinfo&Field=BaseID&Value=HLYX&NaviLink=黑龙江医学)*longjiang Yixue* 1998, **12:**21. | RCT comparing moxibustion on *Zhiyin* combined with self-correction method and chest-knee position to treat breech presentation |
| Yang YK, Mao M, Hu YP, Zhang H, Xu LB, Zhang AR, Gu X, Wang Y, Ao YG, Rong HB: **Effect of moxibustion on Zhiyin point to correct fetus position: multi-center randomized controlled clinical study.** *Zhongyi Zazhi* 2007, **48:**1097-1098. | RCT involving participants of both breech and shoulder presentations |
| Ye DX: **Moxibustion on Zhiyin point combined with Zhuan Tai Prescription for correcting abnormal fetal position in 100 cases**. *Fujian Zhongyiyao* 2004, **35:**24. | CCT involving moxibustion in both groups |
| Zhang QL: **He-Ne laser on ear acupoints for fetal position correction in 50 cases.** *Suzhou Yixue Xuebao* 1996, **16:**755. | RCT comparing laser on acupoint *Zhiyin* and ear acupoints |
| Zhou FX,Zhu XL,Yin SY,Zhu DS,Wu ML,Shi YX: **Effectiveness analysis on three methods for correcting breech presentation.** *Qiqihaer Yixueyuan Xuebao* 1994, **15:**183-184. | RCT comparing among pelvis rotating exercise, recurvation and knee chest position to correct breech presentation |
| Zhou Y, Han YC: **Moxibustion on Zhiyin point combined with breech-raised dorsal position for correcting abnormal fetal position in 42 cases**. *Zhenjiu Linchuang Zazhi* 2005, **21:**41. | RCT involving moxibustion in both groups |
